# Hyperglycemic Patterns in Acute Stroke Patients

**DOI:** 10.7759/cureus.62039

**Published:** 2024-06-10

**Authors:** Antigoni Fountouki, Thomas Tegos, Eleftheria Ztriva, Georgia Kaiafa, Triantafyllos Didangelos, Dimitrios Theofanidis, Christos Savopoulos

**Affiliations:** 1 Nursing, International Hellenic University, Thessaloniki, GRC; 2 1st Department of Neurology, AHEPA University Hospital, Medical School, Aristotle University, Thessaloniki, GRC; 3 1st Propaedeutic Department of Internal Medicine, AHEPA University Hospital, Medical School, Aristotle University, Thessaloniki, GRC; 4 1st Propaedeutic Department of Internal Medicine/Diabetic Care Unit, AHEPA University Hospital, Medical School, Aristotle University, Thessaloniki, GRC

**Keywords:** diabetes type ii, glucose patterns, glucose management, continuous glucose monitoring, stroke

## Abstract

Background and objective

Hyperglycemia following a stroke can independently aggravate the ischemic area. Ensuring adequate glucose management can help avoid complications and minimize mortality and disability in these patients. This study aimed to investigate hyperglycemic patterns in acute stroke patients.

Materials and methods

We conducted a non-interventional prospective observational study involving acute stroke patients by employing continuous glucose monitoring (CGM) for 72 hours after the onset of stroke symptoms. Admission glucose, patients' total mean glucose (TMG), and time in range (TIR) (70-140 mg/dl) were correlated with the hyperglycemic patterns elicited by the CGM system software. Data were analyzed using SPSS Statistics 26.0 (IBM Corp., Armonk, NY) with descriptive statistics, the Kruskal-Wallis test, and χ2 test.

Results

Our cohort comprised 105 diabetic and non-diabetic stroke patients. The hyperglycaemic patterns that we observed were as follows: (i) hyperglycemia from 23:00 to 10:00, (ii) 06.00 to 10.00, (iii) at night and after meals, iv) no pattern, v) unspecified patterns. Patients with nocturnal and morning hyperglycemia had admission glucose of 183 mg/dl, mean 72-hour glucose of 156 mg/dl, and TIR of 37%. Patients who did not develop a hyperglycemic pattern either had admission glucose of 131 mg/dl and TIR of 89% or had high admission glucose (197 mg/dl) and a short TIR (14%). Conventional pre-meal capillary glucose tests do not appear to detect these patients' hyperglycemic tendencies.

Conclusions

These results may indicate the necessity for more intensive measurements during the night or dawn in this patient population. Admission glucose could be considered a predictor of hyperglycemic patterns and contribute to the patient's care plan.

## Introduction

Stroke is a syndrome of acute focal neurological deficit due to vascular injury (infarction or hemorrhage) of the central nervous system with symptoms lasting more than 24 hours [1]. It is the second leading cause of death worldwide. Each year, 13.7 million people are affected by stroke and approximately 5.5 million people succumb to it globally [2]. Approximately 87% of strokes are due to an ischemic infarction [3]. Inadequate blood supply to the local area of the brain causes irreversible tissue damage within minutes in the central core of the damaged area (infarct zone) [4]. However, the surrounding tissue (ischemic penumbra) does not undergo immediate cell death and can recover if early reperfusion is provided [5]. Two-thirds of stroke patients with or without a history of diabetes are reported to present with hyperglycemia, which may be due to the dysregulation of pre-existing or subclinical diabetes or stress-induced hyperglycemia [6].

The American Diabetes Association and the American Association of Clinical Endocrinologists define stress hyperglycemia in hospitalized patients as a plasma glucose concentration >7.8 mmol/l (140 mg/dl) without evidence of pre-existing diabetes [7]. Hyperglycemia aggravates the ischemic brain for several reasons. Firstly, hyperglycemia exacerbates the already existing Ca2+ homeostasis disturbance (due to ischemia). Amino acids, especially glutamate, play a central role in neuronal death by activating postsynaptic glutamate receptors, leading to excessive calcium influx (through ion channels), mitochondrial damage, and eventual cell death. Hyperglycemia may therefore trigger calcium-mediated neuronal death by increasing glutamate availability. Secondly, hyperglycemia promotes anaerobic energy production, resulting in lactic acidosis and further damaging neurons in disadvantaged regions [8].

Several studies suggest that hyperglycemia has an independent effect on ischemic regions, increasing lesion size as well as mortality and disability rates. Even if hyperglycemia is triggered by stress, it can then have a detrimental effect on the ischemic area and cause even greater stress [9]. The frequency of glycemic control depends on the patient's severity and overall condition, previous measurements, and the measures taken as a result; the NICE-SUGAR [10] study argued that a significant number of hypoglycemic episodes occurred in the tight glycemic control group (glucose maintenance target: 81-108 mg/dl). In light of the above, the American Stroke Association has developed guidelines on post-stroke glycemic control and recommends maintaining blood glucose levels in the range of 140-180 mg/dl.

The guidelines generally recommend that patients with diabetes (or hyperglycemia) should have their glucose levels measured every four to six hours, just before a meal, and at bedtime. In patients who take nothing by mouth, or receive continuous enteral nutrition, blood glucose monitoring should be done at regular intervals, usually every six hours, and every one to two hours for people on continuous intravenous insulin or those who are critically ill [11,12,13]. In hospitalized patients, blood glucose monitoring is usually done by traditional capillary glucose testing with a finger stick and glucose meter. Continuous glucose monitoring (CGM) is generally not recommended for hospitalized or critically ill patients, but it can provide sufficient information on the duration of the glucose interval, blood glucose variability [14], and patterns of hyperglycemia during the day or night. Gathering such information can contribute to the development of evidence-based practices and protocols [15,16,17].

This study aimed to examine the patterns of hyperglycemia in patients with acute ischemic stroke by using CGM for 72 hours post-onset of symptoms. Ηyperglycemic patterns are defined as periods of hyperglycemia that recur at roughly the same time intervals during the three-day recording. Some CGM systems automatically calculate these patterns and contribute to the diagnosis, for example, of postprandial hyperglycemia or nocturnal hyperglycemia. Hence, this study aimed to determine the hyperglycemic pattern for evidence-based management of glucose in stroke patients. Guidelines for the frequency of capillary testing were based on a target value of 140-180 mg/dl and the patient's measurement results [10]. In general, it is recommended to test glucose levels before meals and bedtime, and every six hours in patients who do not take anything by mouth or are on continuous enteral nutrition [11].

## Materials and methods

This was a non-interventional prospective observational study that did not involve modifying the clinical management of the patients. The study was conducted at AHEPA University Hospital Thessaloniki, Greece from June 2020 to April 2022. All consecutive patients with ischemic stroke with or without pre-existing diabetes who were admitted to the 1st Propedeutic Department of Internal Medicine of Aristotle University were enrolled in this study. Patients presenting beyond 24 hours from the onset of symptoms were excluded. Other exclusion criteria were as follows: fever, a definite diagnosis of a transient ischemic attack, neoplasm in any organ or tissue within the last five years, and a history of thrombolysis or thrombectomy. The study was completed with 105 patients. Informed consent was obtained from all participants and the study protocol was approved by the Aristotle University Medical Ethics Committee (approval no: 29/7/2020/6.261).

All patients underwent a clinical examination upon admission by an internist and a neurologist, as well as laboratory tests and CT scans as per the applied protocol. A brief mental state test using the Mini-Mental Test was performed to evaluate patients' ability to give consent to participate in the study. To participate, eligible patients were required to provide written informed consent. For patients who did not fulfill this requirement, consent was given by a first-degree relative. CT scans were performed on all patients suspected of having a stroke to rule out transient ischemic episodes. All patients underwent CT with the same CT scanner of the AHEPA hospital. In many cases, the first CT scan failed to detect the infarct, and in such instances, according to the protocol, patients underwent a second scan in the next 24 hours. The first CT that depicted the ischemia was taken into account.

Regardless of infarct imaging findings at the initial CT scan, patients who met the clinical inclusion criteria were fitted with a glucose sensor to perform a three-day blinded CGM. If the second CT scan did not reveal an infarct, the patient was excluded from the study. During the patient's hospitalization, glucose management was performed according to standard clinical practice, which was not affected by the present study. The Medtronic Envision Pro continuous (blind) glucose monitoring system was used. The Envision Pro system is designed to measure glucose levels in interstitial fluid via electrochemical oxidation of glucose and measures glucose every five minutes for up to seven days. The collected data is transferred to a mobile electronic device, using Bluetooth, on which the application is installed. Data is subsequently processed, and the "Patient Report" is assigned to the appropriate website with the use of codes.

CGM measures glucose levels by using a tiny sensor inserted under the skin, usually in the abdomen or upper arm using a special guide. The needle is removed and a few millimeters of capillary catheter remain. It does not require calibration, and hence no capillary measurements were performed on patients for this study, other than those measurements prescribed by the protocol of care and medical guidelines. Glucose data collected by the Envision Pro recorder generates up to five diagnostic reports. The main report, the "Pattern Snapshot", uses advanced recognition algorithms to identify daily patterns of high and low glucose levels and their correlation with food, medications, exercise, and sleep. The Envision Pro CGM system provides blinded recording data that ensures an unbiased view of actual glucose levels whether for the evaluation of a therapeutic intervention or research purposes.

The transmission of the data takes place cumulatively at the end of the monitoring, after the termination of the recording has been activated via the application. The scanning of the sensor from the reading device (mobile phone) can be carried out within seven days even if the sensor has been removed from the patient (e.g., discharge, accidental removal). The parameters recorded for this study were as follows: patients' total mean glucose (TMG), time in range (TIR) (70-140 mg/dl), and the observed patterns. The Kruskal-Wallis test was used to determine if there were statistically significant differences between variables, depending on the satisfaction of the normality condition. The χ2 test was used to investigate the existence of a relationship between two categorical variables. Data were analyzed using SPSS Statistics 26.0 (IBM Corp., Armonk, NY).

The glucose measurements and the overall management of hyperglycemia were performed as per the current international recommendations. Hence, each patient was evaluated by the nursing staff, four times a day via finger-prick glucose measurement. Subcutaneous insulin was administered according to the patient’s needs to achieve a slight hyperglycemic state, i.e., 120-180 mg/dl [18].

## Results

The sample consisted of 105 patients: 44 (41.9%) males and 61 (58.1%) females. The mean age of male patients was 79 years and that of females was 82.2 years; 43 (41%) were diabetic patients. The mean HbA1c and admission glucose of non-diabetic patients were 5.3 and 125 mg/dl, respectively; those of diabetic patients were 6.2 and 177 mg/dl, respectively. TMG was 127 mg/dl (non-diabetics: 112 mg/dl, diabetics: 149 mg/dl). TMG was within range (70-140 mg/dl) in 85% of recording time for non-diabetics and 51% for diabetics (Table 1). Of the 62 non-diabetic patients, six had hyperglycemic TMG with values ranging from 141 to 176 mg/dl, while 41 showed at least one value above 140 mg/dl during the three-day recording. Diabetic patients (n=43) with hyperglycemic TMG were 22, with values ​​ranging from 141-236 mg/dl, and only two of them did not show any hyperglycemic value during the three-day recording.

**Table 1:** Glucose patterns and glucose values on admission SD: standard deviation

Patterns	Admission glucose, mg/dl	N	SD
No pattern in normoglycemic patients	131	60	35.93
No pattern in hyperglycemic patients	197	8	61.75
Hyperglycemia: 23.00-10.00	183	19	86.73
Hyperglycemia: 06.00-10.00	140	9	30.93
Hyperglycemia: at night and after meals	144	3	11.06
Postprandial hyperglycemia	127	3	38.97
Unspecified patterns	118	3	37.07

The hyperglycemic patterns indicate a hyperglycemic tendency in relation to the rest of a 24 hour period. These patterns show that the patient is in a hyperglycemic state throughout the pattern duration. The observed patterns were as follows:

· Incidence of hyperglycemia from 23.00 at night until 10.00 the next morning. This pattern suggests that the patients systematically showed an increase in serum glucose during the night and breakfast hours.

· Hyperglycemia from 06.00 to 10.00 in the morning.

· Hyperglycemia at night and after meals.

· Postprandial hyperglycemia.

· Unspecified patterns.

Patients who did not show any pattern were classified into patients with normal TMG and patients with hyperglycemic TMG.

According to the Kruskal-Wallis test for nonparametric data, glucose on admission showed a dependent association with glucose patterns (chi-square: 23.036, df: 6, p=0.001). Patients with normoglycemia on admission did not show a glucose pattern during three-day glucose monitoring. Eight patients with the highest glucose level on admission with a mean of 196 mg/dl also did not show a hyperglycemic pattern but showed irregular hyperglycemia over a 24-hour period. Patients with medium hyperglycemia (mean glucose: 183 mg/dl) showed a hyperglycemic pattern between 23:00 and 10:00 (during the night and breakfast hours). Nine patients with borderline normoglycemia showed glucose elevation between 6:00-10:00. The remaining patterns manifested in a very small number of patients and concerned postprandial hyperglycemia or other patterns. Also, TMG showed a dependent relationship with the glucose pattern variable (chi-square: 69.878, df: 6, p=0.000) (Table 2). In addition, the variable that shows how much time a patient stayed within the 70-140 mg/dl range (TIR) showed a dependency relationship with the glucose pattern variable (chi-square: 60.122, df: 6, p=0.000) (Table 3).

**Table 2:** Glucose patterns and total mean glucose SD: standard deviation

Patterns	Μean glucose, mg/dl	N	SD
No pattern in normoglycemic patients	109	60	13.841
No pattern in hyperglycemic patients	190	8	27.423
Hyperglycemia: 23.00-10.00	156	19	15.129
Hyperglycemia: 06.00-10.00	123	9	8.746
Hyperglycemia: at night and after meals	136	3	9.644
Postprandial hyperglycemia	141	3	30.414
Unspecified patterns	130	3	21.502

**Table 3:** Glucose patterns and time in range SD: standard deviation; TIR: time in range

Patterns	% TIR	N	SD
No pattern in normoglycemic patients	89	60	17.74
No pattern in hyperglycemic patients	14	8	16.12
Hyperglycaemia: 23.00-10.00	37	19	26.63
Hyperglycemia: 06.00-10.00	81	9	15.26
Hyperglycemia: at night and after meals	58	3	7.57
Postprandial hyperglycemia	57	3	36.17
Unspecified patterns	68	3	28.36

**Figure 1:**
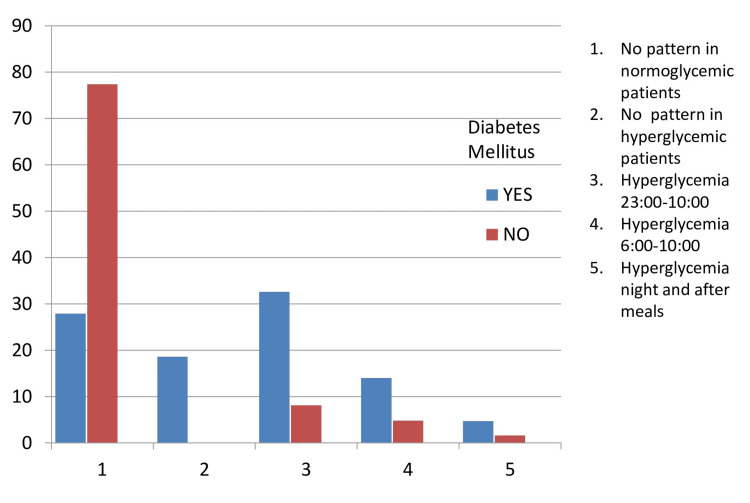
Glucose patterns in diabetics and non-diabetics

Table 4 illustrates the association between glucose patterns and diabetes mellitus (DM). The χ2 test regarding glucose patterns and the presence of DM displayed statistical significance (chi-square: 36.280, df: 6, p=0.000). As depicted in Figure 1, in patients without any pattern and without any hyperglycemia, it was more likely that DM did not exist. On the contrary, in many other patterns, the presence of the disease was observed.

**Table 4:** Glucose patterns and diabetes mellitus DM: diabetes mellitus

Patterns			DM: Yes	DM: No	Total
	No pattern in normoglycemic patients	Count	12	48	60
		% within DM	27.9%	77.4%	57.1%
	No pattern in hyperglycemic patients	Count	8	0	8
		% within DM	18.6%	0.0%	7.6%
	Hyperglycemia: 23.00-10.00	Count	14	5	19
		% within DM	32.6%	8.1%	18.1%
	Hyperglycemia: 06.00-10.00	Count	6	3	9
		% within DM	14.0%	4.8%	8.6%
	Hyperglycemia: at night and after meals	Count	2	1	3
		% within DM	4.7%	1.6%	2.9%
	Postprandial hyperglycemia	Count	0	3	3
		% within DM	0.0%	4.8%	2.9%
	Unspecified patterns	Count	1	2	3
		% within DM	2.3%	3.2%	2.9%
Total		Count	43	62	105

## Discussion

CGM systems have significantly contributed to the management of diabetes and stress hyperglycemia [19]. By performing measurements every five minutes, they have introduced new parameters such as glycemic variability, mean amplitude of glycemic excursions (MAGE), and the mean of daily differences (MODD) [20]. The present study is the first of its kind to utilize another parameter associated with CGMs: hyperglycemia patterns, intending to improve the nursing care plan of stroke patients. Hyperglycemia in stroke has been associated with poor prognosis in both diabetic and non-diabetic patients [21]. Nurses usually perform glucose monitoring, and hence they are in the right position to prevent harmful hyperglycemia. However, this requires clear evidence-based guidelines and protocols [22].

In the present study, of the 61 non-diabetic patients, 41 had at least one hyperglycaemic value during the three-day CGM period. Most non-diabetic patients had sporadic hyperglycemic values apparently due to stress hyperglycemia. Six non-diabetic patients had severe hyperglycemia as their TMG was above 140 mg/dl. The increase in post-stroke glucose is well documented in the critically ill, which is also reflected in this sample, with non-diabetic patients not exceeding the dangerous limit of 180 mg/dl as an overall mean [23].

The hyperglycemic pattern from 23.00 in the night until 10.00 the next morning in 19 patients showed moderate to high hyperglycemia on admission (183 mg/dl). Patients with a mean glucose on admission of 197 mg/dl also did not show a hyperglycemic pattern but revealed unstable high glucose levels, which could not be identified easily, with ordinary standard care. Similarly, in a meta-analysis by Lin et al. (2022) involving stroke patients, glycaemic variability was observed, which again was associated with poorer outcomes [24]. According to our results, the normoglycemic patients with mean glucose of 131 mg/dl on admission exhibited no hyperglycemic pattern, but only sporadic hyperglycemia levels not exceeding 180 mg/dl, which does not warrant a more intensive measurement routine. Moreover, hyperglycemia in non-DM patients during hospitalization is a common clinical finding [25].

The relationship between glucose on admission and glucose patterns is proportional to the relationship between TMG and patterns, but the fluctuations decreased as patients' glucose levels began to be regulated as their hospitalization progressed. Yet, the circumstances that cause nocturnal dysregulation still prevail. It is noteworthy that the early morning values may be due to the "dawn effect", [26] which is also observed in non-diabetic patients. The dawn phenomenon is common in diabetic patients, but, given the effects of stress on stroke patients, whether it leads to very high levels of hyperglycemia needs further investigation. In these lines, DePietro et al. (2016) have also associated high fasting glucose values in the morning with the duration and quality of sleep in hospitalized patients, and indeed, stroke patients’ quality of sleep appears to be disturbed in other studies too [27,28].

Furthermore, patients with stroke may have the ability to swallow safely, but their mental and psychological state may limit their intake to minimal or moderate levels of food consumption; hence, it is difficult to monitor their nutritional status [29]. Nighttime measurements are limited and only performed when patients are highly dysregulated. Finally, in this sample, all patients were fed orally and no patient received an intermittent or systemic parenteral diet. Pre-meal measurements do not appear to detect the pattern of hyperglycemia experienced by patients in this study. While pre-meal measurements are useful for determining the insulin administered, they do not provide information about the increased insulin resistance that stroke patients may experience after meals, at night, and dawn. In our sample, night and morning hyperglycemia usually did not exceed the limit of 180, possibly due to efficient medication regulation or because the specific patients did not show severe dysregulation in DM or stress hyperglycemia. Our results point to a possible nocturnal deviation and this finding should be further studied to be taken into account in the routine nursing care protocol of stroke patients.

Overall, glucose on admission may be a predictive indicator of the course of hyperglycemia and glucose pattern development. Patients with normal glucose levels on admission appear to remain longer within the normal range, with normal TMG but with sporadic hyperglycemic values. Patients with moderate to high hypoglycemia on admission experience improvement in the course of their hospitalization, but show a lower level within the normal range, borderline to high TMG, and nocturnal hyperglycemia patterns. Moreover, patients with normal admission glucose and without DM rarely show any hyperglycemic pattern. In contrast, diabetic patients with hyperglycemia on admission and during their hospitalization are likely to experience hyperglycemia between 11 pm and 10 am

The main strength of this study is its large sample size. However, it has a few limitations, primarily the fact that patients’ comorbidities were not fully taken into account.

## Conclusions

Patients with high glucose on admission exhibit hyperglycaemic trends overnight and at dawn, i.e. at hours when nursing staff does not usually perform blood sugar measurements, except in cases of extreme dysregulation where the treatment plan is individualized. Hence, there is a need for more intense night and afternoon measurements in this population. Overall, since hyperglycemic stroke patients exhibit patterns of hyperglycemia, this should be taken into account when designing care protocols, and CGM systems can contribute significantly in this direction. Yet, as this would require additional measurements, thereby disturbing the patient's sleep, constituting more workload for the staff, and creating extra expenditure on materials, more research is needed to verify the viability of the suggested monitoring scheme in different clinical settings.
